# The efficacy of personalized psychological interventions in adolescents: a scoping review and meta-analysis

**DOI:** 10.3389/fpsyg.2024.1470817

**Published:** 2024-09-06

**Authors:** William Li, John Gleeson, Madeleine I. Fraser, Joseph Ciarrochi, Stefan G. Hofmann, Steven C. Hayes, Baljinder Sahdra

**Affiliations:** ^1^Institute for Positive Psychology and Education, Australian Catholic University, Sydney, NSW, Australia; ^2^School of Behavioral Health Sciences, Australian Catholic University, Sydney, NSW, Australia; ^3^Healthy Brain and Mind Research Centre, Australian Catholic University, Melbourne, VIC, Australia; ^4^Philipps-Universität Marburg, Marburg, Germany; ^5^Psychology Emeritus, University of Nevada, Reno, NV, United States; ^6^Institute for Better Health, Santa Rosa, CA, United States

**Keywords:** adolescent mental health, personalized intervention, psychotherapy, meta-analysis, scoping review

## Abstract

This review compared the efficacy of personalized psychological interventions to standardized interventions for adolescents. We conducted a scoping review and meta-analysis of randomized controlled trials that compared personalized interventions with standardized interventions in adolescents. Data was analyzed using Bayesian multilevel random effects meta-analysis. Eligible studies were identified through five databases: Scopus, PsycINFO, MEDLINE, Web of Science, and EMBASE. Moderation analysis was conducted to explain potential sources of effect size heterogeneity. Eight studies across 13 articles (participant *N* = 2,490) met inclusion criteria for the review with seven studies across 10 articles (*N* = 1,347) providing sufficient data for inclusion in the meta-analysis. A small but significant effect size favoring personalized interventions was found (*d* = 0.21, 95% CrI [0.02, 0.39]), indicating that personalized interventions are associated with superior treatment outcomes compared to standardized interventions. Moderate between-study heterogeneity was found (I^2^ = 53.3%). There was no evidence of publication bias. The review also found significant variation in methods of treatment personalization. This review provides evidence that personalization of adolescent psychological interventions is an effective way to improve treatment outcomes. Given the large number of adolescents worldwide who will experience some sort of mental health problem, personalization could have a significantly large impact on global mental health outcomes.

**Systematic review registration:**

https://doi.org/10.17605/OSF.IO/XRNCG.

## Introduction

Adolescence is defined as a transitional phase from childhood to adulthood that occurs between the ages of 10 and 19 years and is characterized by rapid biological, cognitive, and social change (Cobb, 2010). Studies have shown that the onset of up to 80% of mental health disorders occur before the age of 26 (Caspi et al., 2020), and that between 10 to 30% of adolescents worldwide experience a mental health problem (Kieling et al., 2011; Silva et al., 2020). During this crucial period adolescents who remain free of mental ill health have better long-term outcomes (Caspi et al., 2020). In contrast, an earlier age of onset of mental health problems is associated with an increased risk of persistent mental health disorders into adult life and a greater likelihood of developing comorbid disorders (Caspi et al., 2020).

For most mental health problems, psychological intervention is often recommended as the first-line treatment of choice for adolescents experiencing mental health problems even within the medical field (Pettitt et al., 2022). As a result, over the past five decades, clinical researchers have invested heavily in the development and evaluation of adolescent psychotherapies and there have been considerable efforts to synthesize the evidence for the efficacy of therapies. Indeed, systematic reviews have identified multiple empirically supported interventions for common mental health problems in adolescence including anxiety (Higa-McMillan et al., 2016), obsessive compulsive disorder (Freeman et al., 2014), depression (Weersing et al., 2017), attention-deficit/hyperactivity disorder (Evans et al., 2018), and conduct disorders (Kaminski and Claussen, 2017). A large meta-analysis of 447 studies spanning 30,431 adolescents synthesized 50 years of research on the efficacy of youth psychotherapies found a modest mean post-treatment effect size of only 0.46, with a 63% probability that an adolescent receiving therapy would fare better than control conditions (Weisz et al., 2017). Other meta-analyses have identified smaller study pools that investigated the efficacy of specific adolescent interventions for particular mental health disorders and found similarly modest effect sizes. For example, meta-analyses comparing cognitive behavioral therapy (CBT) to control conditions for anxiety and for depression estimated between group pooled effect sizes of 0.45 (Baker et al., 2021) and 0.41 (Oud et al., 2019), whilst meta-analysis of 14 studies on the efficacy of acceptance and commitment therapy (ACT) for adolescent depression and anxiety found pooled post treatment effect sizes between 0.31 to 0.86 with significant between study heterogeneity (I^2^ = 82.8%; Fang and Ding, 2020).

What is clear from the literature is that current psychological interventions are not effective for all adolescents. Indeed, a recent meta-analysis of 40 studies on psychotherapies for adolescent depression found more than 60% of adolescents did not respond to therapy (Cuijpers et al., 2023). More worryingly it appears that efficacy of empirically supported adolescent psychotherapies is not improving or in some areas is decreasing (Weisz et al., 2019; Jones et al., 2019; Johnsen and Friborg, 2015). This is evidenced by a meta-analysis of 453 studies and 31,993 adolescents spanning over 50 years which found that mean effect sizes of the efficacy of youth psychotherapies have not significantly changed over the past five decades for anxiety or ADHD, and has decreased significantly year after year for depression and conduct problems, with similar effect sizes across passive and active control groups (Weisz et al., 2019).

These findings suggest a worrying trend for clinicians and researchers alike that efforts to improve the general quality of youth psychotherapy models have not translated into improved adolescent outcomes. This pattern of stagnating or decreasing effect sizes, may to some degree, reflect an upper limit to efficacy and growth of current standardized youth psychotherapies. Consequently, there appears to be the need for new approaches to youth psychotherapy design and implementation in contrast to current methods of making small incremental changes to current psychotherapies (Weisz et al., 2019).

One such approach that is re-emerging in the psychological literature is personalization (Ng and Weisz, 2016; Wright and Woods, 2020). Personalization and treatment matching was the norm in the earliest days of evidence-based therapy because it was built into individual functional analysis as a method of diagnosis (Kanfer and Saslow, 1969). Functional analysis as a guide to intervention weakened as syndromal classification became dominant, in large part due to problems of replicability (Hayes and Follette, 1992). There have been increasing calls for its return, based on new and more replicable methods of personalization (Hayes et al., 2020). In the modern era, multiple personalization approaches have been noted in the literature, with treatment-matching and individually tailored approaches being the most prevalent. Treatment-matching involves prospectively matching subgroups of clients to treatments based on hypothesized traits or predetermined methods (e.g., machine learning algorithms, risk factors, etc.; Cohen et al., 2021). This matching can occur within treatment through matching to specific strategies, modules, timings, and dosages (e.g., Delgadillo et al., 2022) or between treatments through matching to a specific treatment package (e.g., Young et al., 2021). Individually tailored approaches involve tailoring treatments to individual clients based on such things as comorbidities, treatment response, or idiosyncratic case conceptualizations (Cohen et al., 2021). Additionally, Collaborative Assessment and Therapeutic Assessment methods are also gaining recognition as personalized approaches, wherein assessment processes are tailored to actively involve clients, thereby aligning interventions more closely with their unique needs and enhancing therapeutic outcomes (Aschieri et al., 2023; Durosini and Aschieri, 2021).

Personalizing interventions is based on the hypothesis that different therapy models, strategies, or components have differing effects on individuals depending on their specific context and characteristics (Wright and Woods, 2020). There is growing evidence to support this hypothesis, as indicated by a recent meta-analysis of clinical trials focusing on psychotherapy for depression. It found a 9% higher variance of treatment effects in intervention groups compared to control groups, suggesting notable heterogeneity in individual responses to therapy (Kaiser et al., 2022). Recent experience sampling method studies provide further evidence of heterogeneity between individuals, with Ciarrochi et al. (2024) finding that processes that were associated with positive outcomes for some individuals were often unrelated or detrimental to others. For example, they found that although 27% of participants benefited from using the strategy of ‘doing things that had worked in the past’, a strategy considered effective at the group level, 14% actually displayed worse mental health outcomes when using this strategy (Ciarrochi et al., 2024). Similarly, Sahdra et al. (2023) found heterogeneity in how individuals relate to self-compassion and compassion for others. They found that higher compassion was associated with greater well-being in individuals who experience self-compassion and compassion for others in harmony (positive correlation), but for individuals whose compassion was not in harmony (uncorrelated or negatively correlated) higher levels of compassion were unrelated to well-being (Sahdra et al., 2023). The findings provide further support for the personalization of interventions as there are clear differences in how different processes and strategies are associated with different outcomes of well-being for different individuals.

There has been growing interest in personalized youth psychotherapy in recent years in both treatment-matching and individually tailored approaches. For example, Höhne et al. (2024) matched adolescent refugees and asylum seekers to four different stepped care interventions based on an individual severity classification. Classification was based on individual depressive symptom severity using the Patient Health Questionnaire with adolescents displaying mild symptoms not receiving an active intervention, adolescents with moderate symptoms receiving a smartphone app developed for use with migrants and refugees, adolescents with moderate to severe symptoms receiving the START_adapt group intervention, and adolescents with severe symptoms receiving individual psychological therapy (Höhne et al., 2024). They found significant reductions in depression and PTSD symptoms with effect sizes of 0.52 and 0.27 respectively, however, found no significant differences between the treatment matched group and treatment as usual control group (Höhne et al., 2024). In contrast, Young et al. (2021) found that adolescents who were matched to either a cognitive behavior program or interpersonal program based on their psychosocial risk (high or low on cognitive and interpersonal risk) showed significantly greater decreases in depressive symptoms than adolescents who were mismatched.

In the realm of individually tailored approaches, modular youth psychotherapies, defined as psychotherapies made up of multiple self-contained and separate modules, have been growing in popularity due in part to their flexibility which facilitates personalization (Ng and Weisz, 2016). A scoping review of decision making in modular treatments for youth found that in 20 different modular youth therapies, 95% recommended using baseline assessment data to make decisions about treatment content, 65% used measurement-based care, and 25% prior research with all therapies recommending using clinical judgment (Venturo-Conerly et al., 2023). There is also evidence to suggest that modular therapies are associated with greater improvements in adolescent well-being outcomes compared to standard empirically supported treatments (Chorpita et al., 2013; Weisz et al., 2012). For example, a study comparing MATCH, a modular youth intervention, and CBT found that the modular approach outperformed CBT on improving internalizing and externalizing symptoms (Weisz et al., 2012). In the study, clinicians in the MATCH treatment group first administered modules related to the problem area defined as most important based on pretreatment assessment measures and client priorities, following this, if an interference arose (e.g., comorbidity, stressors impeding current module, etc.) then the sequence of modules was altered with other modules used systematically to address the interference (Weisz et al., 2012).

As the interest in personalization of youth psychotherapies and interventions is relatively recent, it is yet to be fully established if such personalized psychotherapies and interventions are associated with improved adolescent treatments outcomes when compared to current standardized treatments. In adult populations, a systematic review and meta-analysis on the efficacy of personalized psychological interventions in adults found superior treatment outcomes favoring personalized interventions when compared to both passive control groups and standardized interventions (Nye et al., 2023). Specifically, they found that all studies that compared personalized interventions to passive control groups found superior treatment outcomes favoring personalized interventions, and eight of 14 studies comparing personalized interventions to standardized interventions found superior treatment outcomes favoring personalized interventions (Nye et al., 2023). Further meta-analysis of studies comparing personalized intervention to passive control groups found a large effect size (*d =* 0.89) favoring personalized interventions when compared with passive control groups, whilst meta-analysis of studies that compared personalized interventions to standardized interventions found that that personalized interventions were associated with significantly improved treatment outcomes compared to standardized interventions with a small effect size (*d* = 0.22; Nye et al., 2023). Whilst this effect size is considered small by conventional standards, considering the large population of individuals who engage in psychotherapy, the findings suggest that implementing personalized interventions would still result in a substantial number of individuals experiencing improved treatment outcomes over and above current standardized treatments.

The aim of the current review was to explore the efficacy of personalized psychological interventions in adolescent populations. However, as it is already well documented in the literature that active treatments tend to outperform passive or no-treatment conditions (Penkova et al., 2018; Steinert et al., 2017), and that waitlist control conditions may often over exaggerate the apparent efficacy of interventions (Furukawa et al., 2014; Laws et al., 2022), the current review chose to only focus on studies that compared personalized interventions to active control groups that used a standardized intervention. The aim of the current review was to answer the research questions “are personalized interventions associated with improved psychological well-being and mental health outcomes compared to standardized interventions in adolescents.” In addition, the different methods of personalization, such as the different ways of treatment matching and individually tailoring, and how personalization was achieved, were also investigated. We hypothesized that, similarly to adult populations, personalized psychological interventions would be associated with superior treatment outcomes when compared to standardized interventions in adolescents.

## Method

### Transparency and openness

The protocol for this scoping review, including plans related to the search strategy, data extractions, and analysis, was registered in the Open Science Framework (OSF) database prior to conducting the database search.^1^ There was a minor deviation from the protocol whereby the current review also included studies exploring interventions (personalized vs. standardized) aimed at reducing risk or prevention of early onset of mental health issues in a general population of adolescents (as opposed to adolescents actively seeking treatment for a mental health issue as stated in the protocol). These studies were included on the basis that we believe the findings of these studies contribute to answering the research question “are personalized interventions associated with improved psychological well-being and mental health outcomes compared to standardized interventions in adolescents.” First, we believe these findings are clinically relevant as early intervention or prevention studies often involve similar therapeutic approaches and strategies as those used in treatment studies. Second, we did not limit the treatment setting in the protocol and we believe inclusion of preventative interventions allows for comparison of personalized interventions vs. standardized intervention across different intervention contexts. All data has been made publicly available at the OSF and can be accessed at https://osf.io/4cwpr/.

### Search strategy

The search was conducted in October 2023 using several databases including: PsycINFO, SCOPUS, Web of Science, MEDLINE, and Embase. Key search terms (e.g., personalization, adolescents, RCTs) were combined using Boolean operators (see Supplementary material SA). No restrictions were applied in regard to the date of publication of articles. The inclusion and exclusion criteria were based on similar criteria used by Nye et al. (2023) and altered to match the population of interest of the current review (i.e., adolescents). Similarly to Nye et al. (2023), criteria were developed using a Population Intervention Comparator Outcome Study design framework (PICOS; Table 1) which has been shown to have high sensitivity and specificity compared to other search tools (Methley et al., 2014).

**Table 1:** PICOS framework of inclusion and exclusion criteria.

PICOS	Inclusion criteria	Exclusion criteria
Population	Adolescent clients (mean age between 10 to 19 years old)	Studies where the mean age of participants was under 10 years or over 19 years
Intervention	Studies where participants were prospectively matched to psychological interventions, or where interventions were personalized to the individual participant	Studies which did not prospectively match participants to interventions or personalize the intervention to the individual Personalization only to pharmaceutical treatments Personalization occurs outside of a mental health context (e.g., exercise, diet)
Comparator outcome	Outcome is recorded using a validated patient reported measure, parent reported measure, or therapist reported measure Outcome measure of a psychological construct or related to a mental health issue (e.g., depression, substance use)	Outcome is not recorded using a validated patient reported measure, parent reported measure, or therapist reported measure Outcomes measure is not for a psychological construct or related to a mental health issue (e.g., smoking)
Study design	Study design is a randomized control trial with an active control group receiving a form of standardized intervention (e.g., CBT, psychoeducation)	Study design is not a randomized control trial Randomized control trial does not include an active control group, or it is unclear what the intervention provided is (e.g., waitlist control, usual care with no description of what this involves)

The first and second author screened titles, abstracts, and full texts against inclusion and exclusion criteria. Queries or disagreements were discussed between the two authors and if a decision could not be decided a third reviewer from the research team acted as an intermediary. Studies excluded at the full text screening and reasons for exclusion are outlined in Supplementary material SB. The most common reasons for exclusion were associated with mean participant age (i.e., adult or pediatric populations) and control groups (i.e., no active control group or no standardized intervention control group for comparison). Studies that included a usual care or treatment as usual control group but did not clearly report what interventions these control groups used were excluded as it could not be clearly determined if interventions used in these control groups were standardized evidence-based interventions (e.g., Weisz et al., 2020).

### Data extraction

Data extraction was performed by the first author. The main outcome of interest was whether personalized psychological interventions led to improved psychological well-being or mental health outcomes when compared to standardized interventions. Quantitative data pertaining to primary and secondary outcomes derived from measures of mental health symptoms of wellbeing were extracted at post intervention and follow up time points (where relevant). In addition to statistical outcomes, data related to type of personalization, method of personalization, mental health outcome measure used, treatment duration, treatment delivery method, treatment format, and parental involvement were extracted for use in the planned moderation analysis (Supplementary materials SC, SD).

### Risk of bias assessment

The Cochrane risk-of-bias tool for randomized trials (RoB 2; Sterne et al., 2019) was used to assess risk of bias. The first author conducted the RoB assessment for all included studies, with 50% of included studies (*k* = 6) randomly chosen for independent evaluation by a second rater. Discrepancies were resolved through discussion between first and second raters. The interrater reliability was calculated using Cohen’s kappa statistic, *k* = 0.85, indicating near perfect agreement between raters (McHugh, 2012).

### Data synthesis

A narrative synthesis of treatment outcomes comparing personalized and standardized interventions was conducted on all included studies. Additionally, studies which provided sufficient statistical data were included in a random effects multilevel Bayesian meta-analysis conducted using the *brms* package (Bürkner, 2017) in R. Between group (personalized vs. standardized) effect sizes of treatment by time interactions (treatment outcomes) were calculated using the *esc* package (Lüdecke, 2019) in R and converted to a common metric (Cohen’s *d*) to allow for meta-analysis where needed.

### Bayesian meta-analysis

Bayesian multilevel meta-analysis modeling (Higgins et al., 2009) was used to estimate the overall effect of the efficacy of personalized interventions compared to standardized interventions in adolescents. Bayesian meta-analysis has several advantages over traditional frequentist meta-analytic approaches including superior performance when working with smaller number of studies (Seide et al., 2019), enhanced ability to estimate between study heterogeneity and pooled effect sizes (Seide et al., 2019), and the ability to incorporate prior knowledge and assumptions using prior distributions (Harrer et al., 2021).

For meta-analysis, weakly informative priors, which incorporates weak information on the parameter that covers all possible “real-world” values, without giving any specific value too high of a probability, is recommended (Williams et al., 2018). However, when there is well-supported reason to believe that the parameter falls within a specific range of values, informative priors can be used to enhance precision without compromising accuracy (Morris et al., 2015). The current study used informative priors based on findings from the meta-analysis exploring the efficacy of personalized interventions compared to standardized interventions in adults (Nye et al., 2023) and sensitivity analysis with varying mean priors was also conducted.

When setting a prior for variance, a Half-Cauchy prior is recommended for between-study heterogeneity (*τ*^2^) in a meta-analysis (Williams et al., 2018). In many meta-analyses, *τ* (the square root of *τ*^2^) lies somewhere around the ballpark of 0.3 (Harrer et al., 2021). Consequently, setting the Half-Cauchy prior scaling parameter to 0.3 ensures that a value of less than *τ* = 0.3 has a 50% probability (Williams et al., 2018). However, the current study used a more conservative approach by setting the scaling parameter to 0.5 which flattens the distribution and balances the risks of false positive and false negatives, ultimately leading to more reliable and robust inferences (Harrer et al., 2021; Gelman et al., 2017).

To fit the multilevel model, an intercept-only model with random effects for effect sizes nested within articles nested within studies was specified. Effect sizes were nested within articles to account for the fact that most articles reported several effect sizes for different outcomes, and articles were nested within studies to account for the fact several articles used the same sample of adolescents. Studies with larger sample sizes, and consequently greater precision, were given a greater weight based on the standard error (se) of each effect size (y) in the data, as specified by y|se(se_y) in the following generic formula:


$$y|se\left(se\_y\right)\sim 1+\left(1\mathrm{|Study}/\mathrm{Author}/\mathrm{Effect size}\right)$$

A moderation analysis was conducted to explore potential sources of heterogeneity by incorporating interaction terms in the Bayesian regression models to test whether the effect of the intervention varied according to the levels of the moderator variables. The resulting model was as follows:


$$y|se\left(se\_y\right)\sim 1+\left(1\mathrm{|Study}/\mathrm{Author}/\mathrm{Effect size}\right)+\mathrm{Moderator}$$

Publication bias was explored using Egger’s Regression test, Funnel Plot test, and Trim and Fill method which are recommended as optimal methods of exploring publication bias for the current study’s calculated population effect size and number of included studies (Fernández-Castilla et al., 2021).

## Results

**Figure 1:**
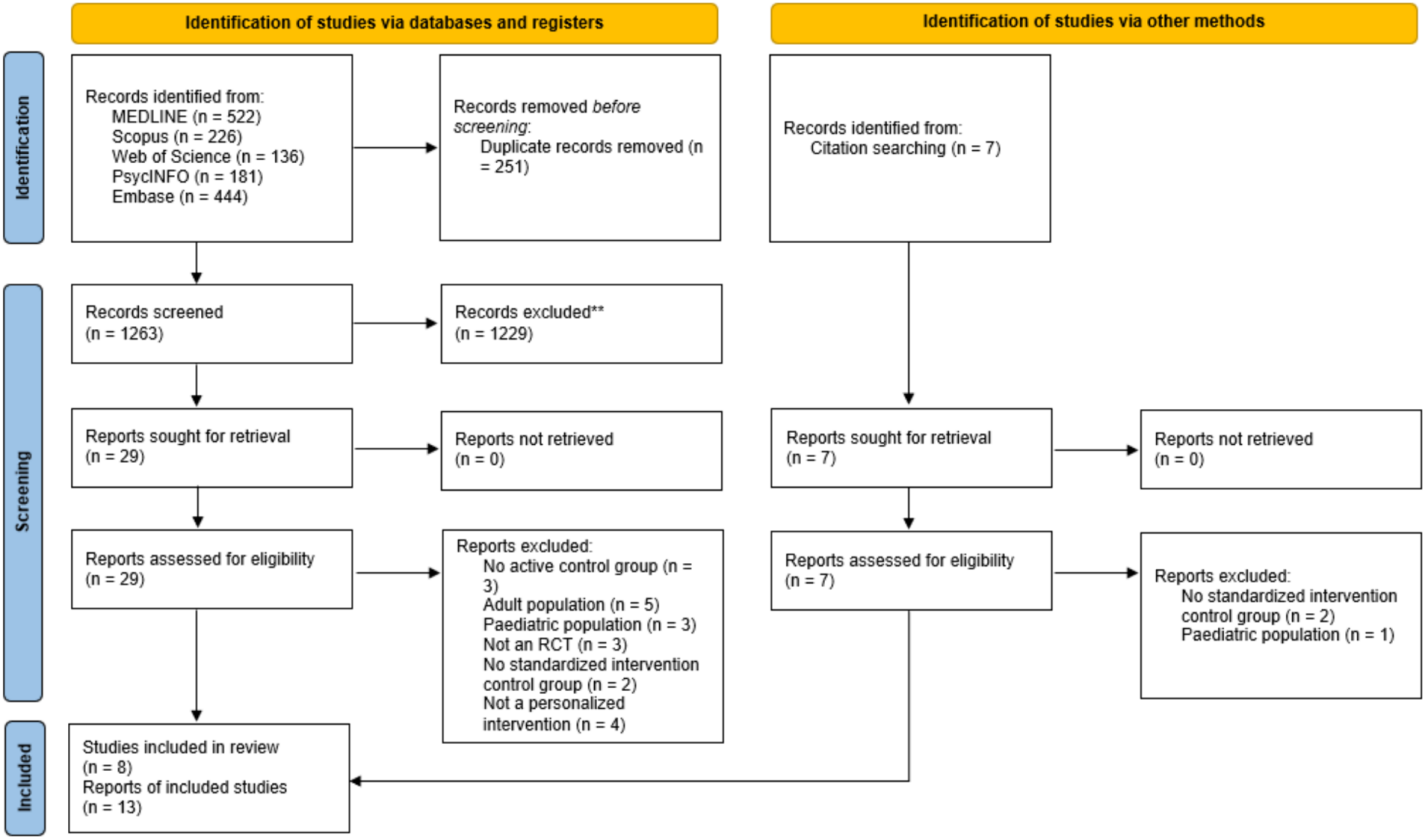
PRISMA flow diagram (Yap et al., 2018).

In total, 13 articles met the inclusion criteria (Figure 1). Across the 13 included articles there were eight separate samples with two studies being follow-up studies (Chorpita et al., 2013; Yap et al., 2019), one study using a subsample of an original sample (Evans et al., 2020), and three studies examining different outcomes in the same sample (Jones et al., 2022, 2023; Young et al., 2021) resulting in a final total of eight included studies. The total number of participants across all eight studies was *N* = 2,490, with sample sizes ranging from 81 to 996 participants. The gender of participants across included studies ranged from 16 to 59% female and mean ages ranged from 10.6 to 18.6 years. Four studies personalized interventions using a treatment-matching approach and four studies examined an individually tailored approach to personalization of interventions. Studies that used a treatment-matching approach to personalization allocated individuals to interventions based on different criteria including drinking motives, symptoms severity, adolescent risk factors, and an algorithm predicting response to treatment. Similarly, studies that used an individually tailored approach to personalization tailored interventions based on different criteria including case conceptualization, response to parenting questionnaires, adolescent risk factors, and a combination of the adolescents’ response to treatment, comorbid problems, and emergence of treatment interfering behaviors. Overall, personalization was achieved by selecting treatment modality (*k* = 2), prescribing specific treatment modules (*k* = 2), prescribing specific strategies/exercises (*k* = 1), selecting treatment intensity (*k* = 1), and providing specific psychological feedback (*k* = 2).

Three studies provided face to face intervention, three studies provided telehealth/online intervention, and two studies provided a combination between face to face and online/app-based interventions. Four studies provided interventions in an individual/one-on-one setting with a trained professional, one study provided intervention in a group setting, one study provided individual online training, and two studies provided interventions in a combination of group and individual settings. One study provided a parent intervention aimed at improving adolescent outcomes, one study provided interventions that involved both the adolescent and a parent, and the remaining six studies provided interventions to the adolescent only. The primary mental health issues examined across studies were substance use (*k* = 2), trauma and depression (*k* = 1), depression (*k* = 1), severe irritability (*k* = 1), and emotional problems (*k* = 1). One study examined depression, dependent stressors, and anxiety across three separate articles, and another study examined a combination of depression, anxiety, and conduct problems and severe irritability across three different articles. In regard to therapeutic approaches used in interventions, the majority of studies used a CBT approach (*k* = 4) with one of these studies using both CBT and Interpersonal Therapy approaches. Other therapeutic approaches used included problem solving (*k* = 1), dialectical behavior therapy and trauma focused CBT (*k* = 1), and person-centered feedback/communication (*k* = 2). Three studies explored primary prevention interventions aimed at reducing adolescent risk and prevention of early onset of mental health issues including depression, emotional problems, and substance use. All included studies compared personalized treatment to standardized treatment whilst six studies also compared personalized treatment to passive control groups.

### Narrative synthesis

Five out of the eight included studies found superior treatment outcomes favoring personalized interventions compared to standardized interventions. Specifically, greater reductions in internalizing and externalizing problems (*k* = 2), substance use (*k* = 2), and anxiety, depressive symptoms, and dependent stressors (*k = 1*). Of the five studies that reported overall superior treatment outcomes for personalized interventions, two studies identified superior treatment outcomes for personalized interventions in only a subsample of participants. Specifically, Wurdak et al. (2015) found reduced substance and alcohol use for personalized intervention only for girls whilst Werch et al. (2010) found that personalized intervention was only associated with reduced alcohol and substance use for adolescents with a history of substance use.

One study (involving three articles) that found overall superior treatment outcomes favoring personalized interventions found no significant differences between personalized and standardized interventions or a slightly superior treatment outcome favoring standardized intervention at post intervention but found significant differences favoring personalized intervention at 18-month follow up (Jones et al., 2022, 2023; Young et al., 2021). Another study that reported superior treatment outcomes for personalized interventions at post intervention also reported 6 month follow up data and found that differences favoring personalized intervention were maintained at follow up (Vivas-Fernandez et al., 2023). In contrast, Weisz et al. (2012) found superior treatment outcomes for personalized intervention at post intervention but when the same sample was assessed at 2 years follow up there were no significant differences in treatment outcomes between personalized and standardized interventions (Chorpita et al., 2013).

Three studies found no significant differences between personalized and standardized interventions. Of these three studies, one study used a treatment matching approach to personalization based on participants’ predicted response to intervention. However, they reported that correlations between participants’ predicted response to intervention for the two different interventions was very high (*r = 0.79*) which may explain their findings.

### Meta-analysis

**Figure 2:**
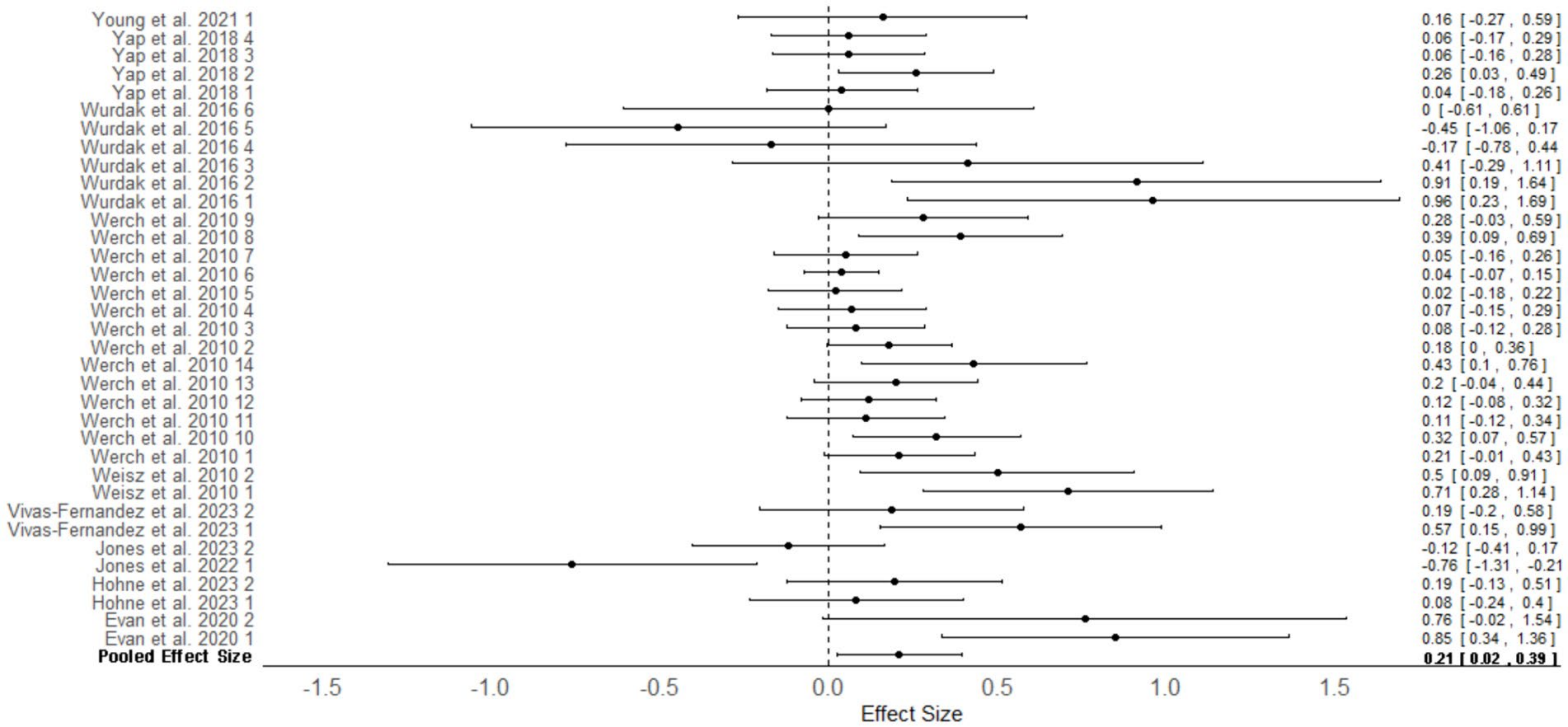
Multilevel random effects meta-analysis: outcomes for personalized intervention versus standardized interventions in adolescents. Numbers next to study authors refers to the effect size number in the article. Some articles reported several effect sizes for differing measures, and these were given a number starting from 1. Effect sizes were nested within articles which were nested within studies.

Seven studies across 10 articles (*N* = 1,347) provided sufficient data to be included in the primary meta-analysis comparing outcomes between personalized interventions versus standardized interventions (Figure 2). The overall mean effect size was Cohen’s *d* = 0.21, 95% CrI [0.02, 0.39], τ (article) = 0.17, 95%CrI [0.01, 0.48], τ (study) = 0.23, 95% CrI [0.03, 0.52], indicating that personalized interventions resulted in superior treatment outcomes relative to standardized interventions in adolescent populations. The overall effect size aggregated a number of mental health and psychological well-being outcomes including depressive symptoms, anxiety symptoms, substance use behaviors, internalizing and externalizing problems, and trauma symptoms. Tests for between-study heterogeneity indicated I^2^ = 53.3%, indicating the presence of moderate between-study heterogeneity (Higgins and Thompson, 2002).

Moderation analysis was performed on the meta-analysis to identify potential sources of heterogeneity in treatment outcomes. This analysis was conducted to help understand which factors might influence the effectiveness of the interventions and explain the variability in results across different studies. The variables examined included the type of personalization, the specific mental health issue addressed, parental involvement, the method of achieving personalization, and whether the intervention was preventative or not.

The results indicated that several factors potentially contributed to the observed heterogeneity (Table 2). Notably, personalized interventions that were individually tailored, those targeting adolescents only, and those that achieved personalization at the component level were associated with superior treatment outcomes compared to standardized interventions. Additionally, personalized interventions demonstrated better outcomes in studies measuring internalizing and externalizing problems, and in cases where the intervention was not preventative but rather targeted ongoing mental health issues in adolescents.

**Table 2:** Moderation analysis results.

Moderator	Sample Size	Effect Size	Credible Interval	I^2
Type of personalization				
Treatment matching	337	0.01	−0.84 to 0.20	63.60%
Individually tailored	1,010	0.32*	0.06 to 0.60	46.60%
Mental health outcome measure				
Anxiety	457	0	−0.41 to 0.38	83.40%
Depression	615	0.01	−0.26 to 0.28	0%
Dependent stressors	98	−0.06	−0.72 to 0.64	N/A
Substance use	441	0.19	−0.35 to 0.77	38.40%
Trauma	158	0.14	−0.36 to 0.66	N/A
Internalizing & Externalizing problems	291	0.56*	0.00 to 1.18	12.70%
Treatment duration				
Brief	441	0.18	−0.21 to 0.57	38.4%
Standard	406	0.42	−0.50 to 0.99	66.40%
Long	497	0.07	−0.77 to 0.53	67.60%
Treatment delivery method				
Face to face	596	0.21	−0.14 to 0.58	60.2%
Online/telehealth	512	0.23	−0.86 to 0.79	30.10%
Hybrid (f2f + online/telehealth)	239	0.18	−0.77 to 0.81	54.20%
Treatment format				
Individual	579	0.28	−0.94 to 0.79	50%
Group	153	0.36	−0.44 to 1.16	44%
Hybrid (group + individual)	256	−0.06	−1.38 to 0.51	62.80%
Parental involvement				
Adolescent only	890	0.30*	0.07 to 0.54	50.9%
Parent only	359	0.09	−0.99 to 0.56	0%
Adolescent & parent	98	−0.19	−1.20 to 0.21	72%
Method of personalization				
Component level	1,091	0.28*	0.08 to 0.49	50.1%
Intensity level	158	0.13	−0.96 to 0.65	0%
Package level	98	−0.19	−1.17 to 0.23	72%
Preventative intervention				
Preventative intervention	611	0.1	−0.77 to0.31	46.60%
Not preventative intervention	736	0.32*	0.01 to 0.65	59.50%

*Denotes statistically significant effect sizes. Component level: Participants are matched to specific treatment modules or components. Intensity level: Participants are matched to specific treatment intensities. Package level: Participants matched to specific treatment packages.

Moderation analysis using Risk of Bias as a variable found that studies rated as having low risk of bias had a mean effect size of *d* = 0.27, 95% CrI [−0.03, 0.59], *I^2^* = 37.8%. Although this was a larger effect size than the primary meta-analysis, it was not statistically significant. Similarly, studies rated as having some concerns yielded a non-significant but smaller effect size of *d* = 0.15, 95% CrI [−0.62, 0.35], *I^2^* = 62.2%.

**Figure 3:**
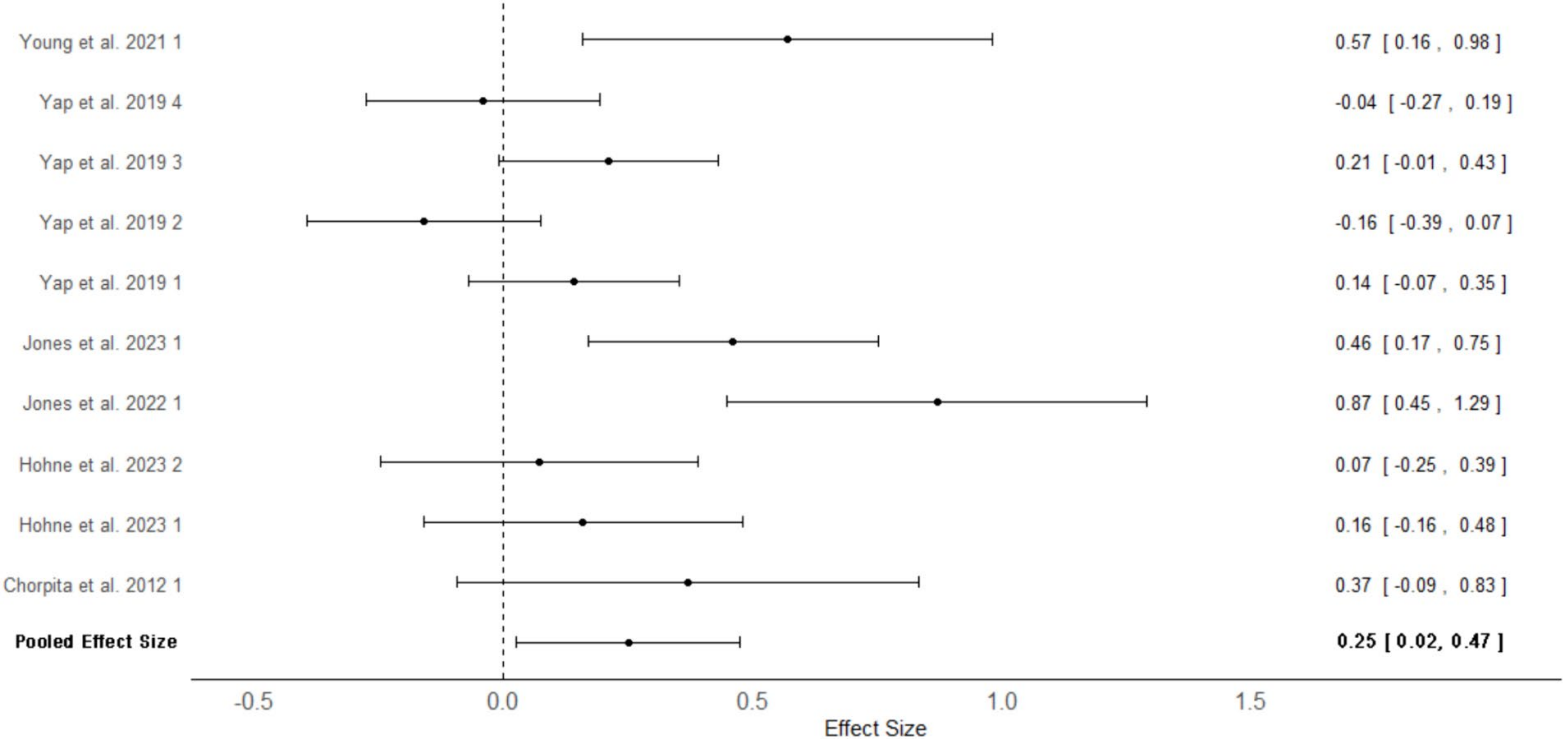
Multilevel random effects meta-analysis forest plot: follow up outcomes for personalized versus standardized Interventions in adolescents. Numbers next to study authors refers to the effect size number in the study. Some studies reported several effect sizes for differing measures, and these were given a number starting from 1. Effect sizes were nested within articles which were nested within studies.

Four studies across six articles (*N* = 950) provided sufficient data to be included in a meta-analysis comparing treatment outcomes for personalized versus standardized interventions at follow-up assessment (Figure 3). The mean effect size was Cohen’s *d* = 0.25, 95%CrI [0.02, 0.47], *I^2^* = 71.2%, indicating that personalized interventions were associated with statistically significant superior treatment outcomes compared to standardized interventions at follow-up. Moderation analysis was also conducted, however, none of the variables were found to explain potential sources of heterogeneity.

### Publication bias

Egger’s Regression test showed a non-significant *p*-value (*p* = 0.17 for primary meta-analysis and *p* = 0.15 for meta-analysis of follow up effects) suggesting the absence of publication bias. This was supported by the Funnel Plot test which showed very minimal funnel plot asymmetry in both plots (Supplementary material SE). Finally, Trim and Fill method found L_o_ = 1.45 for the primary meta-analysis and L_0_ = 0.11 for the meta-analysis of follow up effects which are both smaller than the recommended cutoff of 2 for a meta-analysis with a population effect size of approximately 0.20 and less than 15 studies, suggesting that there is no evidence of publication bias (Fernández-Castilla et al., 2021).

### Sensitivity analysis

Sensitivity analysis was conducted by running several variations of the Bayesian multilevel meta-analysis using differing priors for means. The differing priors included informative priors: μ ∼ *N* (0.22, 0.20); μ ∼ *N* (0.22, 0.30); and μ ∼ *N* (0.22, 0.40), weakly informative priors: μ ∼ *N* (0, 0.12); μ ∼ *N* (0, 0.50), and the non-informative prior μ ∼ *N* (0, 1). Results of the sensitivity analysis are presented in Supplementary material SF. The group level and population effects showed minimal change across differing priors suggesting the results reported are relatively robust. However, the 95%CI for the intercept of the weakly informative and non-informative priors included zero, whilst the majority of the informative priors did not. These findings that different mean priors led to different interpretations of the model suggest that the results may not be stable. However, the similarity of the estimates across models somewhat decreases this concern (Reis et al., 2023). Overall, sensitivity analysis revealed that the results of the meta-analysis are slightly dependent on prior selection. Further sensitivity analysis was conducted by removing the nesting of articles in studies and instead combining articles that reported on the same sample as one article or study in the meta-analysis with following model:


$$y|se\left(se\_y\right)\sim 1+\left(1\mathrm{|Article}/\mathrm{Effect size}\right)$$

The resulting group level and population effects showed minimal to no change (Cohen’s *d* = 0.21, 95% CrI [0.06,0.36], τ (article) = 0.24, 95% CrI [0.09, 0.49]) suggesting robustness in the multilevel model used.

## Discussion

This scoping review and meta-analysis aimed to explore the efficacy of personalized psychological interventions compared to standardized non-personalized interventions for adolescents. The results indicated that adolescents who received personalized psychological interventions display superior treatment outcomes compared to adolescents who received standardized non-personalized interventions. The effect size for personalized interventions versus standardized interventions was Cohen’s *d* = 0.21, which is considered a small effect size (Cohen, 1988). This effect size was nearly identical to the effect size of Cohen’s *d* = 0.22 found in adults (Nye et al., 2023) suggesting that personalized interventions may have similar superiority compared to standardized treatments in adolescent and adult populations. More specifically, personalized interventions were found to be associated with superior treatment outcomes compared to standardized interventions in measures of internalizing and externalizing problems with a medium effect size (*d* = 0.56).

Similar to findings in an adult population, individually tailored approaches to personalization were associated with superior outcomes compared to standardized interventions (*d* = 0.32). However, in contrast to adults, treatment-matching approaches were not associated with superior outcomes compared to standardized interventions in adolescents. A possible explanation may be that several articles that used a treatment-matching approach only found superior treatment outcomes favoring personalized intervention at follow-up but not post-intervention (Young et al., 2021; Jones et al., 2022, 2023). Although these articles reported findings from the same sample of adolescents it indicates the possibility that the benefits of personalized interventions may take time to manifest and hints at the longer-term positive effects of personalized intervention.

Regarding how personalization was achieved, component level personalization (matching to specific treatment modules or components) was associated with superior treatment outcomes compared to standardized interventions (*d* = 0.23). However, intensity level and package level personalization methods were not associated with improved outcomes compared to standardized interventions. In adults, component level personalization was found to be a particularly effective method of personalization (*d* = 0.37) compared to other personalization methods (Nye et al., 2023) and the findings from the current meta-analysis suggest similar findings in adolescents. These findings suggest that rather than targeting personalization on a larger scale such as selection of which therapy modality or treatment package to use or the intensity of treatment, that personalization on a smaller scale targeting the components or kernels of an intervention may be more effective at improving treatment outcomes.

Personalized interventions that targeted the adolescent only (compared to parent only or adolescent and parent interventions) were found to be associated with superior treatment outcomes compared to standardized treatments with a small effect size (*d* = 0.30), suggesting that personalization is most effective when personalization is focused solely on the adolescent accessing treatment. Interestingly, this finding is contrary to the literature which suggests that adolescent interventions involving parents generates significantly better outcomes in reduction of psychopathology than interventions involving the adolescent only (Pine et al., 2024). A possible explanation may be that studies in the current review that involved parents in treatment targeted internalizing problems which have been found to not significantly benefit from parental involvement (Pine et al., 2024). Furthermore, unclear boundaries around confidentiality when there is parental involvement in adolescent treatment has been found to trigger negative reactions and hinder treatment (Calabrò et al., 2024). Although articles in the current review did not report on how confidentiality was managed in parent and adolescent interventions, this may possibly also explain this interesting finding. However, it must be noted that the small number of studies and sample size of interventions that involved both adolescents and parents in the current review potentially underestimates the true impact of parent-inclusive interventions. Personalized interventions were also associated with superior treatment outcomes compared to standardized interventions when providing treatment for an ongoing mental health issue (as opposed to a preventative intervention) with an effect size of *d* = 0.32. This was larger than the effect size of the primary meta-analysis (*d* = 0.21) and suggests that personalization is most effective and best used when designing interventions for adolescents experiencing ongoing mental health issues. In contrast, adolescents receiving interventions aimed at primary prevention of mental health issues may see fewer benefits from personalization.

Personalized interventions were also found to be associated with superior treatment outcomes compared to standardized interventions at follow-up (*d* = 0.25), indicating that improved treatment outcomes of personalized interventions compared are maintained for up to six to eighteen months post intervention. A core goal of psychological intervention is to cultivate improvement in mental health and psychological functioning which is maintained across time. This goal is even more important for adolescents, as such changes may adjust the long-term mental health trajectory of the individual into adulthood. Therefore, the current meta-analysis lends strong support for the benefits of personalizing psychological interventions for adolescents.

In terms of methods of personalization, there was significant variation across studies in how personalization was implemented. Methods ranged from using adolescent risk factors determined prior to intervention, case conceptualization, parent questionnaires, drinking motives, and machine learning algorithms. The findings indicated that there is currently no superior method for achieving personalization with many varying methods currently available. This is supported by findings by Bastiaansen et al. (2020) who provided an individual client’s experience sampling methodology data to 12 different psychology research teams and asked for recommended personalized treatment targets. Interestingly, they found significant variation in how teams analyzed the data, the types of statistics used, and the rationale for targeting the same treatment targets indicating that selection of personalized treatment targets is still highly conditional on subjective analytical choices (Bastiaansen et al., 2020).

### Future research

Across included studies, only one study used a statistical data driven model of personalization (Ahuvia et al., 2023), and only one study achieved personalization at the intensity level (Höhne et al., 2024). Consequently, it is still unclear if these forms of personalization could lead to superior treatment outcomes compared to standardized interventions in adolescents. Further investigation of these methods would be beneficial. Data driven statistical and machine learning models of personalization incorporate experience sampling methodologies, allowing for intensive repetitive assessment of an individual in their everyday natural environment. These methods may be a particularly promising area for further research given their replicability and the possibility of data-driven algorithmic improvement, perhaps driven by artificial intelligence and machine learning procedures. This method can also readily link personalization to idionomic analysis of known processes of change, which is a direction that is receiving increased attention (Hayes et al., 2022) as a modern empirical form of process-based functional analysis (Hayes et al., 2020).

### Strengths and limitations

The strengths of the current review included the pre-registration of the study protocol, the large number of searched databases, risk of bias analysis with reliability checks, and use of citation searches. Another strength was the use of multilevel Bayesian meta-analysis using priors based on results from a meta-analysis on the efficacy of personalized interventions compared to standardized interventions in adults. The use of a multi-level meta-analysis allowed for consideration of different outcomes measures nesting within articles and articles nesting within studies due to several studies using the same sample. Finally, sufficient studies were available to conduct a secondary meta-analysis exploring the efficacy of personalized interventions compared to standardized interventions at follow-up assessment to explore if benefits of personalization are maintained over time.

The current study also had several limitations. First, although the meta-analysis yielded a significant effect size favoring personalized intervention, this finding is somewhat dependent on the priors for means used in the analysis. Consequently, these findings need to be updated as additional research exploring personalization of adolescent interventions emerges, and priors from the current study could be used in future studies and reviews to expand the current findings. Second, due to the relatively limited number of studies included in the meta-analysis of follow-up effects, a more comprehensive moderation analysis to explore potential sources of heterogeneity could not be conducted. Third, the main findings mixed together a wide variety of specific problem areas and outcomes, which may disguise more specific domain-dependent effects. As the impact of personalized interventions may vary across different problem areas, pooling results from a diverse range of issues could lead to a more generalized estimate of effectiveness. While this provides a valuable overall perspective, it may slightly obscure the precise impact of personalized interventions in specific contexts. Consequently, while the overall findings remain robust, they might not fully capture the nuances of effectiveness for each unique problem area and highlights the potential for further exploration into specific problem domains. Finally, the exclusion of grey literature and studies not written in English, the small number of included studies, and that data extraction was performed by one reviewer should be considered.

### Clinical implications

The results of this meta-analysis indicate that in adolescent populations, personalized interventions are associated with superior treatment outcomes compared to standardized non-personalized interventions. Although the calculated effect size of Cohen’s *d* = 0.21 is considered a small effect by conventional standards (Cohen, 1988), given the increasingly large number of adolescents experiencing a mental health issue and accessing treatment (Racine et al., 2021), a significantly large number of adolescents may benefit from improved outcomes of personalized interventions. Indeed, this effect size equates to a number needed to treat of NNT = 8.47, meaning approximately 1 in 8 adolescents would experience improved treatment outcomes if personalized interventions were implemented. This is extremely similar to findings in adult populations (NNT = 8.50; Nye et al., 2023) indicating that personalization could benefit a large proportion of the global population.

In that context it is worth noting that despite the central role of personalization in the earliest days of the evidence-based therapy movement, actual research on the topic is still somewhat limited, and a number of potentially important avenues of personalization remain to be explored. Thus, the small but meaningful effect size found across both adult and adolescent areas should be thought of as an initial benchmark that strongly justifies further research, but not necessarily a ceiling for what might be possible. We do not know if increased research attention will improve the effect size for personalization, but given the practical and theoretical centrality of the issues it raises, it is time to find out.

The results of the moderation analysis suggest that using an individually tailored approach may be more effective than treatment matching adolescents to specific treatment packages. Specifically, individually tailoring specific components or modules of treatment to suit the needs of the adolescent receiving treatment appears to be the most appropriate and effective method of personalizing interventions. Personalized interventions also appear to be most effective when personalization is focused on the adolescent receiving treatment for an ongoing mental health issue. Finally, personalization of adolescent interventions may have long-term benefits given it is likely to be early in the course of these disorders.

## Conclusion

Personalized psychological interventions for adolescents are associated with superior treatment outcomes compared to standardized treatments. These benefits favoring personalized treatments also appear to be maintained at follow-up assessments. These findings indicate that the efficacy of adolescent psychological care could be improved by adopting a personalized approach to intervention.

## Data Availability

The original contributions presented in the study are included in the article/Supplementary material, further inquiries can be directed to the corresponding author.

## Footnotes

^1^ https://osf.io/xrncg
