# Supplementary material for: The efficacy of personalized psychological interventions in adolescents: a scoping review and meta-analysis
Source: Front Psychol. 2024 Sep 6;15:1470817. doi: 10.3389/fpsyg.2024.1470817 (PMC11413809; doi:10.3389/fpsyg.2024.1470817)
Supplement: Supplementary file 1 [file Data_Sheet_1.PDF]

Supplementary Materials A:

Search Terms

Personali?ed OR individuali?ed OR tailored OR “treatment match*” OR “treatment selection” OR stratif*

AND

Psychotherap* OR intervention* OR therap* OR treatment

AND

adolescen* OR youth OR teen* OR “school student*”

AND

“Mental health” OR "mental disorder" OR depress* OR anxiety OR stress OR trauma OR “eating disorder” OR OCD OR “attention deficit” OR “personality disorder” OR substance

AND

RCT OR randomi?ed

Supplementary Materials B:

Studies Excluded at Full Text Screening

Author	Year	Title	DOI	Reason for Exclusion
Andersson et al.	2017	Interactive voice response with feedback intervention in outpatient treatment of substance use problems in adolescents and young adults: A randomized controlled trial	10.1007/s12529-016-9625-0	No active control group
Berger et al.	2014	Internet-based guided self-help for several anxiety disorders: a randomized controlled trial comparing a tailored with a standardized disorder-specific approach	10.1037/a0032527	Adult population (Mean age above 19 years)
Blevins & Stephen	2016	The impact of motives-related feedback on drinking to cope among college students	10.1016/j.addbeh.2016.02.024	Adult population (Mean age above 19 years)
Boyer et al.	2016	Qualitative treatment-subgroup interactions in a randomized clinical trial of treatments for adolescents with ADHD: Exploring what cognitive-behavioral treatment works for whom	10.1371/journal.pone.0150698	No standardized intervention control group
Buckner et al.	2020	On-line personalized feedback intervention for negative affect and cannabis: A pilot randomized controlled trial	10.1037/pha0000304	No active control group
Chorpita et al.	2017	Child STEPs in California: A Cluster Randomized Effectiveness Trial Comparing Modular Treatment With Community Implemented Treatment for Youth With Anxiety, Depression, Conduct Problems, or Traumatic Stress	10.1037/ccp0000133	Paediatric population (Mean age under 10 years)
Ebesutani et al.	2016	A Pilot Study of Modular Cognitive-Behavioral Therapy and Cognitive-Behavioral Hypnotherapy for Treating Anxiety in Iranian Girls	10.1521/ijct_2016_09_01	No standardized intervention control group
Foster et al.	2019	Estimating patient-specific treatment advantages	10.1016/j.jpsychires.2019.02.021	Not a personalized

		in the ‘Treatment for Adolescents with Depression Study’		intervention
Gewirtz et al.	2019	Does giving parents their choice of interventions for child behavior problems improve child outcomes?	10.1007/s11121-018-0865-x	Paediatric population (Mean age under 10 years)
Gunlicks-Stoessel et al.	2019	Latent Profiles of Cognitive and Interpersonal Risk Factors for Adolescent Depression and Implications for Personalized Treatment	10.1007/s10802-019-00552-3	Not a personalized intervention
Gunlicks-Stoessel et al.	2016	An adaptive treatment strategy for adolescent depression: Beginning with NBSP; interpersonal psychotherapy for depressed adolescents	10.1016/j.jaac.2016.07.586	Not an RCT
Han et al.	2023	Using Machine Learning To Identify Biopsychosocial Factors And Predict Treatment Outcomes In A Randomized Controlled Trial For Youth With Chronic Pain	10.1016/j.jpain.2023.02.271	Not an RCT
Huibers et al.	2015	Predicting Optimal Outcomes in Cognitive Therapy or Interpersonal Psychotherapy for Depressed Individuals Using the Personalized Advantage Index Approach	10.1371/journal.pone.0140771	Adult population (Mean age above 19 years)
Langer et al.	2022	Shared decision-making for youth psychotherapy: A preliminary randomized clinical trial on facilitating personalized treatment	10.1037/ccp0000702	No standardized intervention control group
Paz Castro et al.	2022	Longer-Term Efficacy of a Digital Life-Skills Training for Substance Use Prevention	10.1016/j.amepre.2022.06.017	No active control group
Rapee et al.	2017	Comparison of stepped care delivery against a single, empirically validated cognitive-behavioral therapy program for youth with anxiety: A randomized clinical trial	10.1016/j.jaac.2017.08.001	Not a personalized intervention
Santisteban et al.	2011	Preliminary results for an adaptive family treatment for drug abuse in Hispanic youth	10.1037/a0024016	Not a personalized intervention
Storch et al.	2022	Moderators of response to personalized and	10.1007/s10803-021-05000-0	Paediatric population

		standard care cognitive-behavioral therapy for youth with autism spectrum disorder and comorbid anxiety		(Mean age under 10 years)
Vaiva et al.	2018	Combining Postcards, Crisis Cards, and Telephone Contact Into a Decision-Making Algorithm to Reduce Suicide Reattempt: A Randomized Clinical Trial of a Personalized Brief Contact Intervention	10.4088/JCP.17m11631	Adult population<br>(Mean age above 19 years)
van Bronswijk et al.	2021	Precision medicine for long-term depression outcomes using the Personalized Advantage Index approach: cognitive therapy or interpersonal psychotherapy?	10.1017/S0033291719003192	Adult population<br>(Mean age above 19 years)
Walton et al.	2022	ADAPTIVE INTERVENTIONS FOR ALCOHOL USE AND VIOLENCE AMONG YOUTH IN THE EMERGENCY DEPARTMENT: A SMART STUDY	10.1111/acer.14831	Not an RCT
Weisz et al.	2020	Testing Robustness of Child STEPs Effects with Children and Adolescents: A Randomized Controlled Effectiveness Trial	10.1080/15374416.2019.1655757	No standardized intervention control group
Wood et al.	2021	Modular Cognitive Behavioral Therapy for Autism-Related Symptoms in Children: A Randomized Controlled Trial	10.1037/ccp0000621	Paediatric population<br>(Mean age under 10 years)

Supplementary Materials C:

Primary Outcomes of Included Studies.

Study	Total N	Analysed N	Narrative outcome	Statistical outcome
Wurdak et al. (2016)	81	Personalised = 32<br>Standard = 49	For girls, those who received a motive tailored/individualised intervention reported lower drinking frequency and less binge drinking at follow up than girls who received standard intervention.<br>For boys, there was no difference between the two groups.	Personalised vs Standardised time x group interaction.<br>For Girls:<br>Frequency of alcohol consumption: F = 7.770, p = 0.009<br>Frequency of binge drinking: F = 7.005, p = 0.013<br>Frequency of drunkenness: F = 1.414, p = 0.243<br><br>For Boys:<br>Frequency of alcohol consumption: F = 0.310, p = 0.581<br>Frequency of binge drinking: F = 2.150, p = 0.150<br>Frequency of drunkenness: F = 0, p = 0.988
Hohne et al. (2023)	158	TM = 79<br>TAU = 79	No significant differences between treatment matched group and treatment as usual group in both depression and PTSD symptoms.	No significant difference between TM and TAU: F (02,150) = 0.27, p = 0.762 for depression (PHQ).<br>No significant difference between TM and TAU: F (2,127) = 1.48, p = 0.230 for PTSD symptoms (CATS).
Werch et al. (2010)	416	Tailored = 179<br>UC = 181<br>Tailored (drug use history) = 55	No significant differences between any measures of alcohol, cigarette, or marijuana use between tailored and UC.<br>However, for adolescents with a history of	Effect sizes (Cohen's d):<br>Alcohol frequency: 0.21<br>Alcohol quantity: 0.18<br>Alcohol heavy use: 0.08

		UC (drug use history) 46	drug use. Significant differences in drug/alcohol use problems favouring tailored interventions. Also showed reduced frequency of alcohol use, and heavy alcohol use in favour of tailored interventions.	Marijuana frequency: 0.07<br>Marijuana quantity: 0.02<br>Marijuana heavy use: 0.04<br>Substance use problems: 0.05<br><br>Substance use history subgroup.<br>Alcohol frequency: 0.39<br>Alcohol quantity: 0.28<br>Alcohol heavy use: 0.32<br>Marijuana frequency: 0.11<br>Marijuana quantity: 0.12<br>Marijuana heavy use: 0.20<br>Substance use problems: 0.43
Vivas-Fernadez et al. (2023)	208	Personalised = 52<br>Standard = 54<br>Control (UTALK) = 47	Comparison between PROCARE (standardised) and PROCARE+ (personalised) found that PROCARE+ was significantly superior in the reduction of level of emotional risk and separation anxiety reported by parents with small effect sizes. Post-hoc comparisons between ACC (standardised) and PROCARE + found that PROCARE+ was associated with significant improvements in most primary outcome measures.	ACC vs PROCARE +: X^2 (2, N = 99) = 7.42, p = 0.02.<br><br>PROCARE and PROCARE+: X^2 (2, N = 106) = 0.92, *p* = 0.62.
Jones et al. (2023)	98 (original sample 204)	Matched = 50<br>Unmatched = 48	Matched and mismatched adolescents did not differ on rates of change in dependent stressors during the intervention (i.e., from baseline to post-intervention). However, matched adolescents showed significantly greater reductions in dependent stressors from	Matched vs mismatched post intervention t = -.81, *p* =.42.<br>Matched vs mismatch 18month f/u t=3.17, *p* = .002 Cohen's *d* = 0.46 [0.17, 0.74]

			baseline to 18 months f/u compared to mismatched adolescents.	
Jones et al. (2022)	98 (original sample 204)	Matched = 50<br>Unmatched = 48	Matched adolescents showed minimal reductions in anxiety symptoms relative to mismatched adolescents at post intervention. From post-intervention to 18-month follow-up matched adolescents showed a decrease in anxiety symptoms whereas mismatched adolescents showed a significant increase in symptoms.	Matched vs mismatched 18month f/u, d = 0.87, p =.001<br>Matched vs mismatched post intervention, d = -0.76, p = .01 (- negative indicates superior outcomes for mismatched youth)
Young et al. (2021)	98 (original sample 204)	Matched = 50<br>Unmatched = 48	Matched adolescents showed significantly greater decreases in depressive symptoms than mismatched adolescents from postintervention through 18-month follow-up and across the entire 21-month study period. But no significant differences in depressive symptoms at post intervention.	Matched vs mismatched post intervention d = 0.16, p = 0.41<br>18-month f/u d = 0.57 CI [0.16, 1.02]
Ahuvia et al. (2023)	996	Lucky = 7<br>Unlucky = 1	Only eight participants had PAI scores above 0.5 SD: seven ‘lucky’ participants and one ‘unlucky’ participant. Consequently, did not have sufficient data to conduct a t-test comparing outcomes by luck among participants with clinically significant PAI scores. Among all participants (including those whose PAI scores were below 0.5 SD), there were not significant differences in actual RTI between ‘lucky’ participants and ‘unlucky’ participants.	Lucky/matched: M=0.66, SD=1.11<br>Unlucky/mismatched: M= 0.60, SD=1.09<br>Comparison: t=0.44, p= .656

Weisz et al. (2012)	174	Standard Manualised Treatment (SMT) = 69<br>MATCH (Personalised/Modular treatment) = 69<br>Usual Care (UC)= 64	The findings showed significantly steeper trajectories of improvement in the MATCH intervention than in the SMT intervention on most primary and secondary measures.<br>Also found significant steeper improvement of symptoms in the MATCH intervention than in the UC treatment condition on moist primary and secondary measures.	Effect sizes:<br>MATCH treatment outperformed UC treatment.<br>BPC total: 0.59 *p* =.004<br>TPA total: 0.54 *p* = .011<br><br>MATCH treatment outperformed SMT.<br>BPC total: 0.71 *p* =.001<br>TPA total: 0.61 *p* = .012
Chorpita et al. (2013)	174	SMT= 69<br>MATCH= 70<br>UC= 64	2-year follow up of Weisz et al. (2012).<br>Rate of improvement of internalizing and externalizing symptoms was not significantly different between the modular/personalised (MATCH) intervention and standardised intervention (SMT). However, MATCH was associated with a significant advantage over UC during the 2-year assessment period, whereas the treatments in the SMT group was not.	Effect Sizes:<br>MATCH vs STM (Total overall problems) = 0.37<br>MATCH vs UC (Total overall problems) = 0.65<br>MATCH vs UC (Youth report) = 0.45<br>MATCH vs UC (Caregiver report) = 0.59<br>MATCH vs STM (Youth report) = 0.29<br>MATCH vs STM (Caregiver report) = 0.11
Evans et al. (2020)	174 (93 subsample of SIMD)	SMT = 26<br>MATCH = 26<br>UC = 24	Among adolescents with SIMD, those in the MATCH intervention showed statistically significant and reliable within-group improvement per all measures and both informants (parent and adolescent). In contrast, UC and SMT both tended to show within-group improvement on caregiver-reported measures but not consistently on all youth-reported measures. MATCH also produced faster rates of improvement relative to UC or SMT, or both. Finally, MATCH and SMT both led to meaningful reductions in	Effect Sizes:<br>MATCH vs SMT BPC total (Caregiver)= 0.85<br>MATCH vs SMT BPC total (Youth) = 0.76

			functional impairment relative to UC, however, only MATCH predicted significantly fewer diagnoses at post-treatment.	
Yap et al. (2018)	359 (parent child dyad)	PiP = 179<br>Control = 180	No significant interactions between condition and time on the SMFQ-P, SMFQ-C, and SCAS-P scores. Across both conditions (personalised and standardised), parents reported significantly decreased symptoms of depression and anxiety from baseline to post intervention.	Effect sizes (Cohen's d):<br>SCAS-P: 0.04 (−0.18 to 0.26)<br>SCAS- C: 0.26 (0.03-0.48)<br>SMFQ-P: 0.06 (−0.16 to 0.28)<br>SMFQ-C: 0.06 (−0.17 to 0.28)
Yap et al. (2019)	359 (parent child dyad)	PiP = 179<br>Control = 180	No indication that PiP (personalised intervention) significantly reduced adolescent depression and anxiety symptoms as reported by either parents or adolescents, compared to standardised intervention at 12-month follow up.<br>Consistent with findings at postintervention, suggesting that the effect of PiP was not associated with significant reductions in adolescent symptoms compared with a standardised intervention.	Effect sizes (Cohen's d):<br>(Negative scores indicate greater reduction in scores in personalised intervention group)<br>SCAS-P = −0.14 (−0.37 to 0.08)<br>SCAS-C = 0.16 (−0.07 to 0.39)<br>SMFQ-P = −0.21 (−0.42 to 0.01)<br>SMFQ-C = 0.04 (−0.19 to 0.27)

Supplementary Materials D:

Characteristic of Included Studies

Study	Country	Setting/delivery format	Participants	Mental Health Issue	Intervention and control group	Personalisation	Primary Outcome Measure	Secondary Outcome Measures
Wurdak et al. (2016)	Germany	Hospital<br><br>F2f/online hybrid<br><br>Individual	N=81, Mean age = 15.6 years, SD = 1.0, 42% female	Alcohol use (adolescents hospitalised due to alcohol intoxication)	HaLT intervention + motive-tailored exercises based on evaluated treatment programs or psychological theories (eg., dealing with stress, interpersonal skills, etc.)<br>HaLT intervention + general exercises.	Treatment Matching (drinking motives)	Alcohol consumption (adapted from the ESPAD)	N/A
Hohne et al. (2023)	Germany	Outpatient/Community sample<br><br>F2f/online hybrid<br><br>Group/individual hybrid	N=158, Mean age = 18.6 years, SD = 1.58, 16% female.<br>Refugees or asylum seekers.	Depression/Trauma	Level 1- Watchful waiting (PHQ score 5-9)<br>Level 2 – Smartphone app "Balsam" (PHQ score 10-14)<br>Level 3 – Group intervention "START adapt" (PHQ score 15-19)<br>Level 4 – Psychotherapy (PHQ score 20-17)<br>TAU – Psychological support, medication, medical support.	Treatment Matching (Based on symptom severity)	Primary Health Questionnaire (PHQ-9)	Child and Adolescent Trauma Screen (CATS)
Werch et al. (2010)	USA	School health promotion project<br><br>F2f<br><br>Individual	N=416, Mean age = 15.8years, SD = 0.77, female 63.5%	Substance use (alcohol, cigarette, marijuana) (preventative)	Brief Image-Based Intervention+ Tailored in person communication involving screening survey, consultation, goal plan).<br>UC – commercially available health promotion materials	Individually Tailored	Health and Personal Development Survey<br><br>17 item alcohol and drug problems (Costa et al., 1999).	
Yap et al.	Australia	Secondary	N = 332	Depression and	Partners in Parenting	Individually	Parenting to	Short

(2018)		schools/communit y<br><br>Online/web based<br><br>Individual	adolescents (359 parents), Mean age = 13.7 years, 44.5% female (child)	anxiety	Intervention (PiP) – web-based parenting program with individually tailored feedback reports.<br>UC – Educational Factsheets	Tailored	Reduce Adolescent Depression and Anxiety Scale (PRADAS)	Mood and Feelings Questionna ire (SMFQ)<br><br>Spences Children's Anxiety Scale (SCAS)
Yap et al. (2019)	Australia	Secondary schools/communit y<br><br>Online/web based<br><br>Individual	N = 332 adolescents (359 parents), Mean age = 13.7 years, 44.5% female (child)	Depression and anxiety<br><br>12-month f/u of Yap et al. (2018)	Partners in Parenting Intervention (PiP) – web-based parenting program with individually tailored feedback reports.<br>UC – Educational Factsheets	Individually Tailored	Parenting to Reduce Adolescent Depression and Anxiety Scale (PRADAS)	Short Mood and Feelings Questionna ire (SMFQ)<br><br>Spences Children's Anxiety Scale (SCAS)
Vivas-Fernadez et al. (2023)	USA	Community<br><br>Telehealth<br><br>Group	N=208, Mean age = 13.71 (SD = 1.41), Female 48.5%	Emotional Problems (preventative)	PROCARE Intervention vs PROCARE+ (PROCARE INTERVENTION + additional models tailored according to adolescent risk factor determined pre-intervention)	Treatment Matching (based on risk)	Strength and Difficulties Questionnaire (SDQ)<br><br>10-Item Connor-Davidson Resilience Scale (CD-RISC-10)<br><br>KIDSCREEN-10 Index	Difficulties in Emotion Regulation Scale (DERS)<br><br>Willingnes s and Action Measure for Children and Adolescent

								s (WAM-C/A)<br><br>Revised Child Anxiety and Depression Scale (RCADS-30)
Jones et al. (2023)	USA	Community<br><br>F2f<br><br>Individual/Group hybrid	N = 204, Mean age = 14.26 (SD = 1.65), 56.4% female	Depression (preventative)	Coping with Stress (CWS) Interpersonal Psychotherapy - Adolescent Skills Training (IPT-AST)	Treatment Matching (Based on risk)	Adolescent Life Events Questionnaire (ALEQ)	N/A
Jones et al. (2022)	USA	Community<br><br>F2f<br><br>Individual/Group hybrid	N = 98, Mean age = 13.93 (SD = 1.67), Female 59%	Depression (preventative)	Coping with Stress (CWS) Interpersonal Psychotherapy - Adolescent Skills Training (IPT-AST)	Treatment Matching (Based on risk)	Multidimensional Anxiety Scale for Children (MASC)<br><br>Schedule for Affective Disorders and Schizophrenia for School-Age Children – Present and Lifetime Version (KSADS-PL)	N/A
Young et al. (2021)	USA	Community<br><br>F2f<br><br>Individual/Group	N = 204, Mean age = 14.26 (SD = 1.65), 56.4% female	Depression (preventative)	Coping with Stress (CWS) Interpersonal Psychotherapy - Adolescent Skills Training (IPT-AST)	Treatment Matching (Based on risk)	Schedule for Affective Disorders and Schizophrenia for School-	N/A

		hybrid					Age Children – Present and Lifetime Version (KSADS-PL)<br><br>Children's Depression Inventory (CDI)	
Ahuvia et al. (2023)	USA	Community, Single Session Intervention (SSI)<br><br>F2f<br><br>Indivdual	N = 996 overall (but only 7 matched and 1 mismatched after algorithm prediction)	Depression	Project Personality (PP) Action Bring Change (ABC) Project	Treatment Matching (Based on response to treatment prediction algorithm)	Childrens Depression Inventory 2nd Edition Short Form (CDI-2-SF)	N/A
Weisz et al. (2012)	USA	Community treatment<br><br>F2f<br><br>Individual	N=174, Mean age = 10.59 (SD = 1.76) years, range 7 -13 years old, 30% female	Various (depression, anxiety, conduct, etc.)	MATCH, Standardised treatment (Coping Cat for anxiety, PASCET for depression, Defiant Children for disruptive behaviour)	Individually Tailored	Brief Problem Checklist (BPC)<br><br>Top Problems Assessment (TPA)	Children's Interview for Psychiatric Syndromes (Child and Parent versions)
Chorpita et al. (2013)	USA	Community treatment<br><br>F2f<br><br>Individual	N=174, Mean age = 10.59 (SD = 1.76), 30% female<br><br>(Same sample as Weisz et al., 2012) - 2 year follow up.	Various (depression, anxiety, conduct, etc.)	MATCH, Standardised treatment (Coping Cat for anxiety, PASCET for depression, Defiant Children for disruptive behaviour)	Individually Tailored	Child Behaviour Checklist (CBCL)<br><br>Youth Self-Report (YSR)<br><br>Brief Impairment Scale (BIS)<br><br>Services Assessment	N/A

							for Children and Adolescents–Brief Parent Version(SACA)	
Evans et al. (2020)	USA	Outpatient mental health treatment<br><br>F2f<br><br>Individual	N=174, Mean age = 10.6years, SD = 1.8, range 7-13 years, 30% female	Severe irritability	MATCH, Standardised treatment (Coping Cat for anxiety, PASCET for depression, Defiant Children for disruptive behaviour), Usual Care	Individually Tailored	Child Behaviour Checklist (CBCL) or Youth Self Rating (YSR), Brief Problem Checklist (BPC)	ChIPS, BIS, Youth top problems, BPC

Supplementary Materials E:

Funnel Plot for Primary Meta-Analysis Exploring Personalised Interventions versus Standardised Interventions.

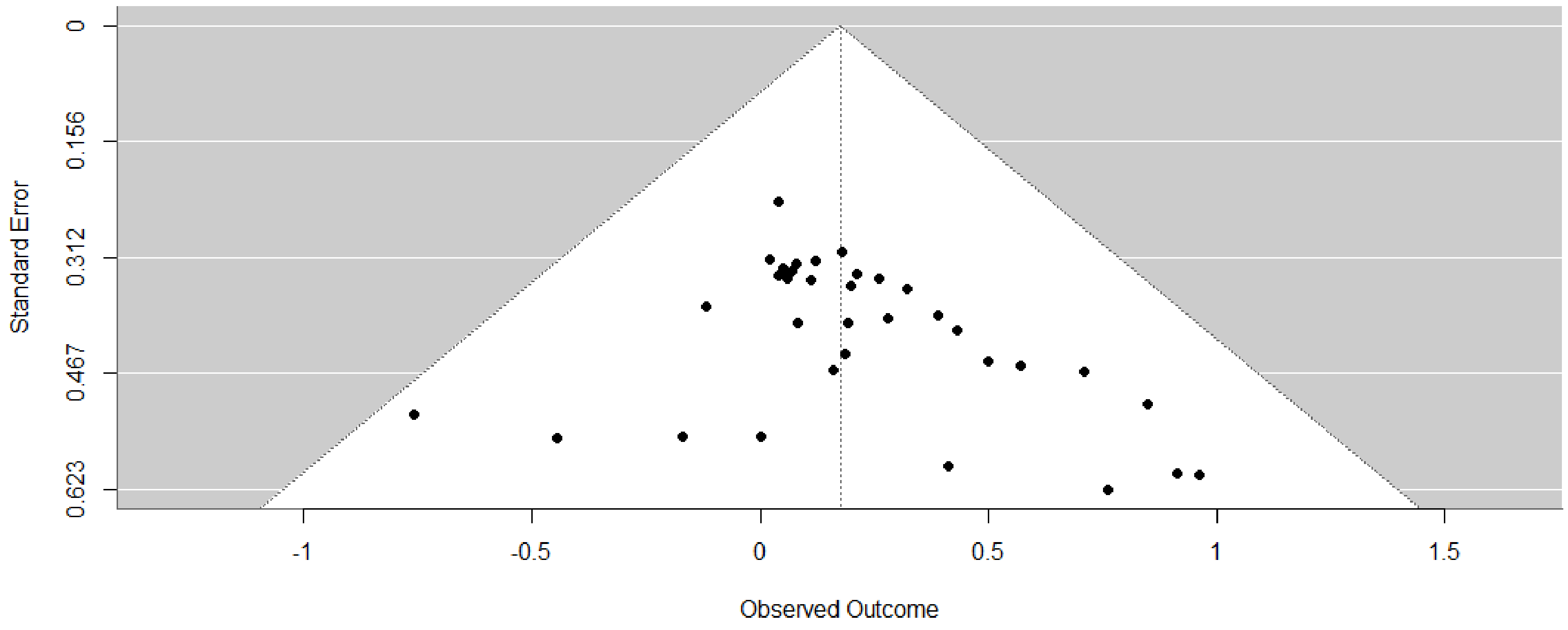

Funnel Plot for Secondary Meta-Analysis Exploring Personalised Interventions versus Standardised Interventions at Follow-up.

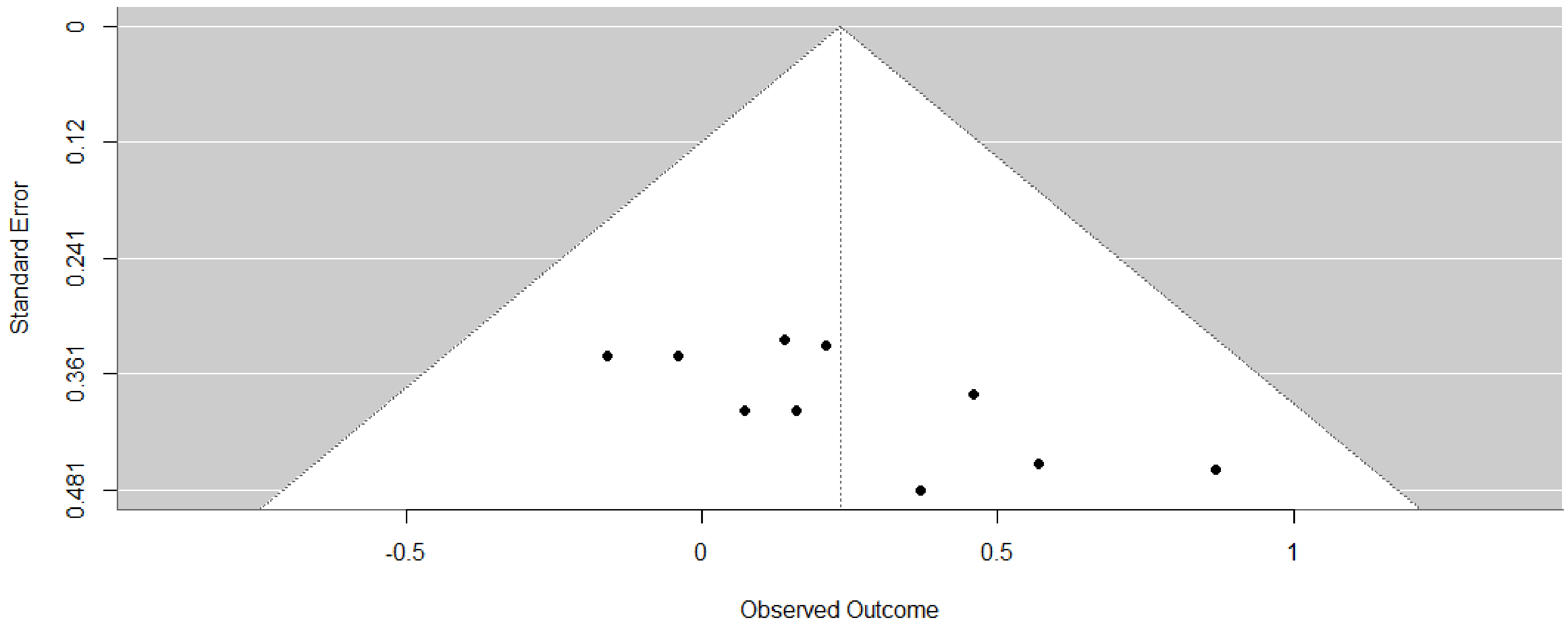

Supplementary Materials F:

Effects sizes estimates, standard errors (SE), and Credible Intervals for primary Bayesian meta-analysis using different means priors.

Priors	Sample (SE)	Sample/Author (SE)	Sample/Author/Measure (SE)	Population Level Effects (SE)	Credible Intervals
μ ~ N(0.22, 0.20)	0.23 (0.13)	0.18 (0.13)	0.06 (0.04)	0.20 (0.10)	0 to 0.41
μ ~ N(0.22, 0.30)	0.24 (0.13)	0.18 (0.13)	0.06 (0.04)	0.20 (0.12)	-0.03 to 0.44
μ ~ N(0.22, 0.40)	0.24 (0.13)	0.18 (0.13)	0.06 (0.04)	0.20 (0.12)	-0.05 to 0.45
μ ~ N(0, 0.12)	0.24 (0.13)	0.19 (0.14)	0.06 (0.04)	0.10 (0.09)	-0.09 to 0.26
μ ~ N(0, 0.50)	0.24 (0.13)	0.18 (0.13)	0.06 (0.04)	0.18 (0.13)	-0.08 to 0.44
μ ~ N(0, 1)	0.24 (0.14)	0.18 (0.14)	0.06 (0.04)	0.19 (0.13)	-0.07 to 0.46
